# Novel attributes of cell‐free plasma mitochondrial DNA in traumatic injury

**DOI:** 10.1002/ctm2.1055

**Published:** 2022-10-17

**Authors:** Grant T. Daly, Viktor M. Pastukh, Yong B. Tan, C. Michael Francis, C. Zack Aggen, S. Chris Groark, Carson Edwards, Madhuri S. Mulekar, Mohammad Hamo, Jon D. Simmons, Matthew E. Kutcher, Emily M. Hartsell, Darrell L. Dinwiddie, Zachary M. Turpin, Hank W. Bass, Justin T. Roberts, Mark N. Gillespie, Raymond J. Langley

**Affiliations:** ^1^ Department of Pharmacology University of South Alabama College of Medicine Mobile Alabama USA; ^2^ Department of Surgery University of South Alabama Colleges of Medicine Mobile Alabama USA; ^3^ Department of Physiology and Cell Biology University of South Alabama College of Medicine Mobile Alabama USA; ^4^ Department of Mathematics and Statistics University of South Alabama Colleges of Medicine and Arts and Sciences Mobile Alabama USA; ^5^ Department of Surgery University of Mississippi School of Medicine Jackson Mississippi USA; ^6^ Department of Pediatrics University of New Mexico School of Medicine Albuquerque New Mexico USA; ^7^ Department of Biological Science, College of Arts and Sciences Florida State University Tallahassee Florida USA

Dear Editor,

Plasma mitochondrial DNA (mtDNA) fragment abundance has emerged as a biomarker in multiple human disorders, thus pointing to the prospect that mtDNA, like nuclear DNA (nDNA), could be a useful substrate for liquid biopsy.^1^, ^2^, ^3^, ^4^ Structural attributes of plasma mtDNA fragments, may contain prognostic information beyond quantitative‐polymerase chain reaction (PCR) measured abundance.^5^, ^6^ While deep sequencing could be informative, the method is limited by the low concentration of mtDNA relative to nDNA in plasma.^7^ In this communication, we describe a combined target‐bait enrichment, sequencing and analytical protocol to improve quantitation and structural insights into plasma mtDNA fragments. Plasma obtained on hospital admission from 30 consecutive patients admitted to a single academic surgical‐trauma intensive care unit was utilized to determine if mtDNA damage‐associated molecular patterns (DAMP) abundance or other parameters were associated with acute complications.

Attributes of mtDNA DAMPs enriched from plasma utilizing a commercially available target‐bait capture kit were explored using Next Generation sequencing on an Illumina platform. We then developed an alignment and filtering strategy to fully quantify mtDNA DAMP abundance over the entire mitochondrial genome, characterize fragment lengths, and identify mtDNA heteroplasmy. (Figure 1A).

**FIGURE 1:**
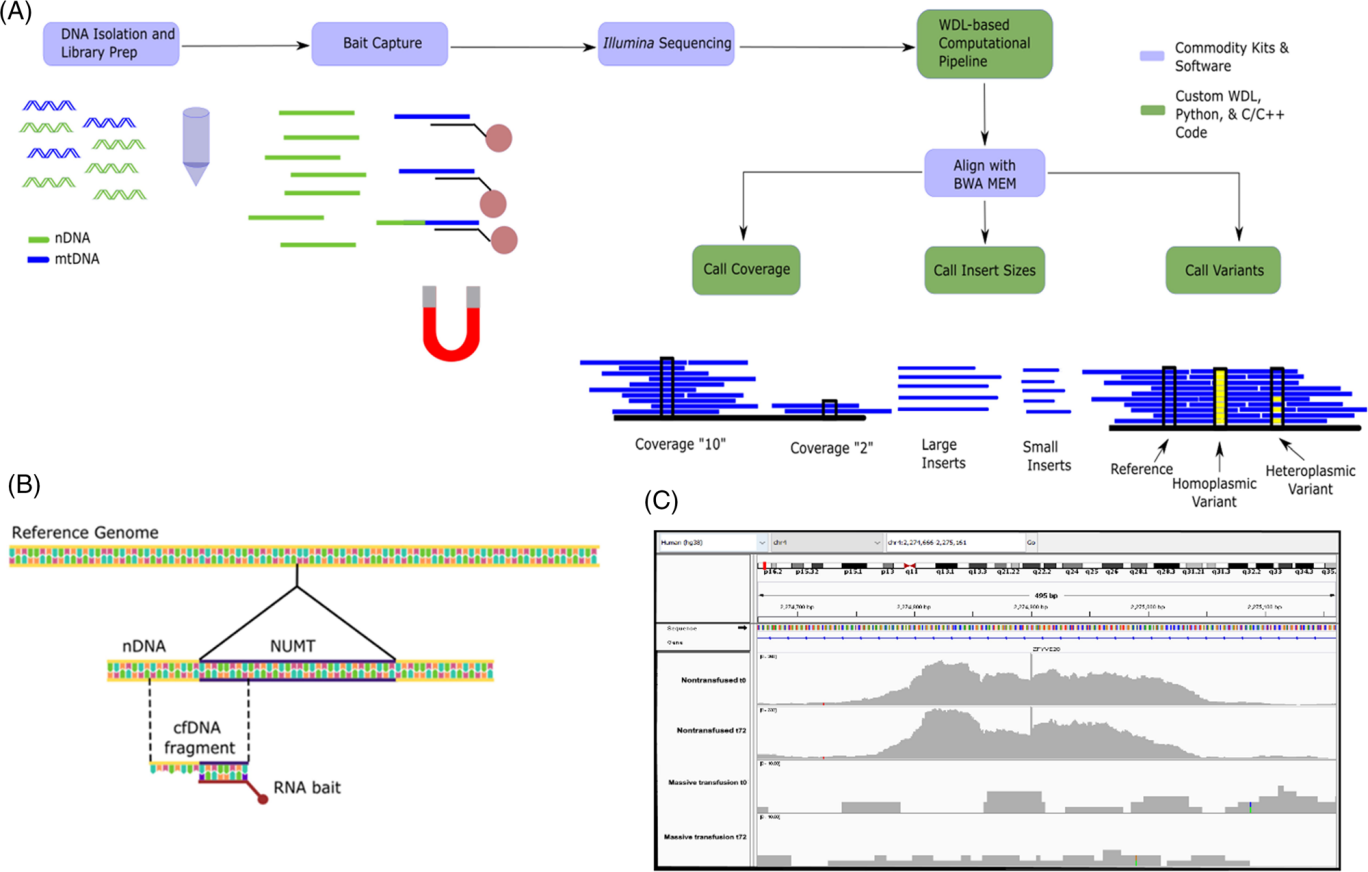
**RNA target bait‐capture and bioinformatics protocol and nuclear mitochondrial (NUMT) identification**. (A) DNA is isolated from plasma or tissue. In the figure, nuclear DNA (nDNA) and mitochondrial DNA (mtDNA) are denoted by colour. DNA isolation and library preparation are applied to all sample DNA, regardless of nuclear or mitochondrial origin. A target bait‐capture kit consists of biotinylated RNA probes complementary to the mitochondrial genome. The probes efficiently bind mtDNA but can also bind homologous NUMT, as illustrated by the DNA fragment half coloured as mitochondrial and half coloured as nuclear. Once enriched, samples are pooled and sequenced on a standard Illumina instrument. From there, a Workflow Description Language pipeline aligns the reads to the whole genome –nuclear and mitochondrial. Three custom C/C++ programs built on the htslib library then call mitochondrial and nuclear coverage, insert (the size of a fragment after end repair and sequencing adapter ligation), and variant calling. (B) Schematic depiction of how target‐bait capture also leads to the sequencing of flanking regions of polymorphic NUMTs. (C) Integrated Genome Viewer (IGV) histograms depicting a specific polymorphic NUMT in a nontransfused patient whereas the second patient lacks this insertion at either t0 or t72 post‐admittance. Subfigures (A) and (B) were prepared in Inkscape. (C) Prepared from IGV.

Due to high sequence homology, we assumed the target‐bait capture method would also enrich nuclear mitochondrial (NUMT) pseudogenes.^8^ There are two varieties of NUMTs; those enumerated in the reference genome, and a second that is polymorphic, meaning they are found sporadically in the population.^8^ About 1500 reference NUMTs have been identified, spanning ∼100 Kbp of the nuclear genome. A simulation analysis of mitochondrial sequences and NUMT sequences determined that while most reads were uniquely aligned, there were multiple reads that align with both the mitochondrial and nuclear genomes (Figure S1A–C). While quality scores >20 improve mapping efficiency, some reads cannot be uniquely aligned. Therefore, to improve accuracy, our algorithm judiciously excludes all reads that ambiguously align to both nuclear and mitochondrial genomes. While this potentially undercounts mtDNA DAMP abundance, it improves the rigour of variant classification.

Detection of polymorphic NUMTs is far more challenging than the enumerated NUMT population because the latter often share greater than 99% homology, are not contained in the reference assembly, and can only be discovered by matching paired reads that align to both the nuclear and mitochondrial genomes.^8^ As depicted, polymorphic NUMTs captured by target‐bait enrichment can be identified by sequenced fragments of nDNA outside of the NUMT insertion point (Figure 1B). Some read pairs aligned to both the nuclear and mitochondrial genomes, while the regions flanking the polymorphic NUMT insertion sites were not homologous to the mtDNA genome (Figure 1C). There is currently no available strategy that can completely eliminate polymorphic NUMTs. However, because of the apparent low frequency of this type of NUMT, their inadvertent inclusion is unlikely to lead to a significant overestimation of the authentic mtDNA DAMP abundance.

In four patients from a protocol development study, we found that the enrichment and alignment strategy produced 1412 ± 1333 (mean ± SD)‐fold enrichment of mtDNA as compared to WGS (Figure 2A). We explored the utility of our protocol by characterizing plasma mtDNA DAMPs in 30 consecutively‐enrolled trauma patients (Figure 2B). First, to determine if there were differences in mtDNA DAMP abundance or fragment lengths as a function of the mtDNA sequence from which they aligned, we normalized these parameters into 100 bp bins and depicted means ± S.D. as a function of the bin from which they originated, finding no sequence‐dependent differences (Figure 2C,D). Thus, in this cohort and patient population, quantitation of mtDNA DAMP abundances or fragment lengths determined at any point spanning the mitochondrial genome should be equally informative. It remains to be determined whether the same will hold true for other disorders. A considerable number of total heteroplasmies were identified ranging in prominence from comparatively rare to homoplasmy (Figure 2E). These heteroplasmic variants tended to cluster around the d‐loop region and sporadically at other sites across the mitochondrial genome.

**FIGURE 2:**
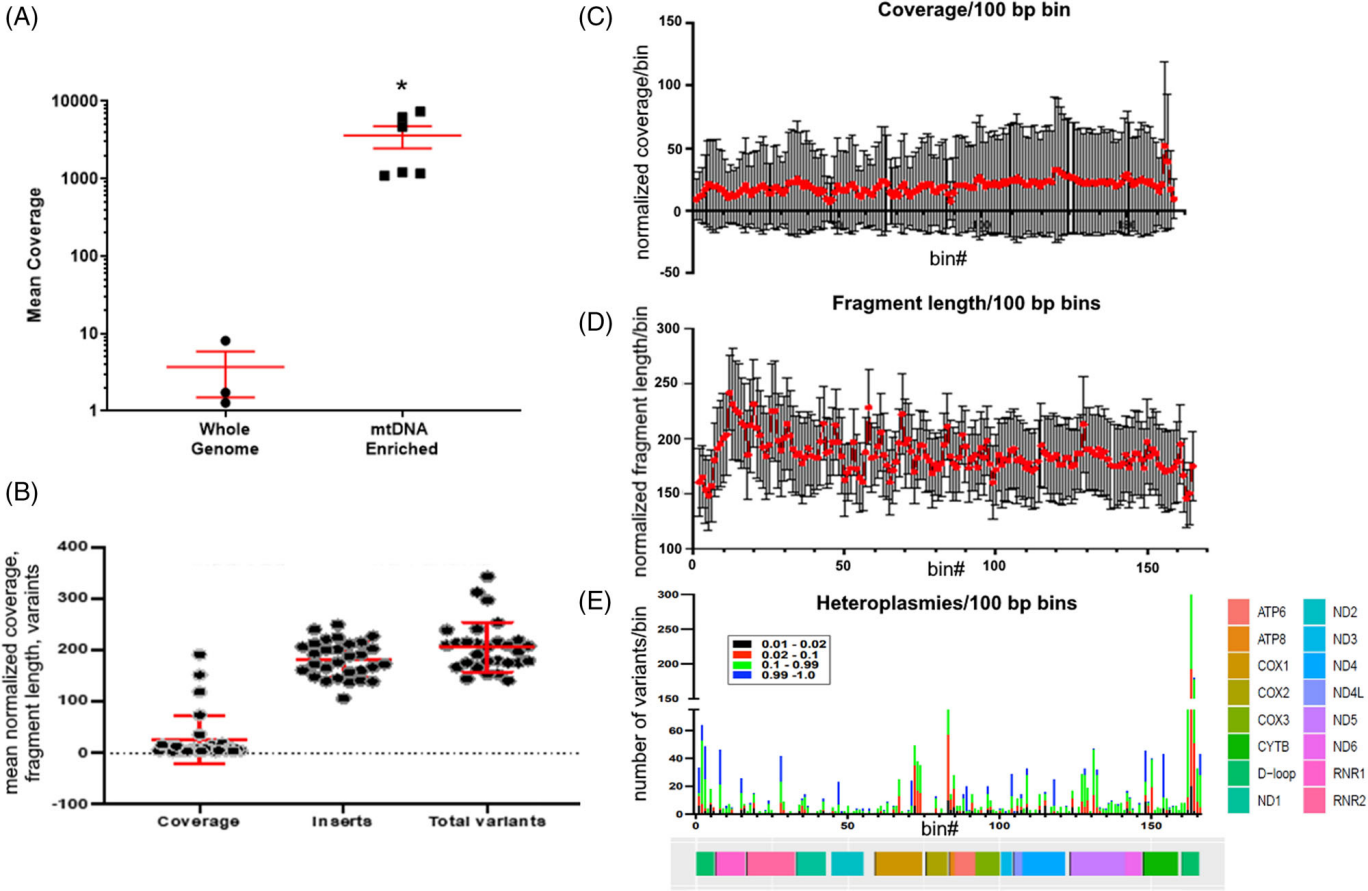
**Enrichment, mean genome‐wide coverage, fragment length and total variants in trauma patients**. (A) Target‐bait capture enrichment in combination with the judicious exclusion of nuclear mitochondrial (NUMT) leads to significant enrichment of the mitochondrial DNA (mtDNA) genome versus whole genome sequencing of cell‐free plasma. Enrichment efficiency was calculated for mean coverage of the mtDNA genome (reads/base) after whole genome sequencing and after target‐bait enrichment in four individual patients. Mean ± standard error of the mean; *p ≤ .05. (B) Normalized mean coverage of mtDNA (mean coverage/mean NUMT coverage), fragment length (mean length, measured in bp) and a number of heteroplasmies for all 30 trauma patients. Hundred base pair nonoverlapping bins of (C). normalized mean coverage across the mtDNA genome and (D) mean fragment length across the ≈16.5 kb expanse of the mitochondrial genome. (E) Heteroplasmic variants in all 30 patients as reported in 100 bp bins, where colour demarcates the variant allele fraction (VAF) of the variants. See “methods” for details.

Next, we examined associations between plasma mtDNA DAMP abundance, fragment lengths and heteroplasmic variant signatures with injury severity score (ISS), systemic inflammatory response syndrome (SIRS), acute kidney injury (AKI) and acute lung injury (ALI). Figure 3A‐D shows that, in the current cohort of trauma patients, mtDNA DAMP abundance failed to discriminate between patients with non‐severe (ISS ≤ 14) or severe (ISS ≥ 15) injuries as defined on hospital admission, or SIRS, AKI and ALI classified 72 h after admission. In contrast, mtDNA DAMP fragment length was significantly shorter in patients with ISS ≥15 compared to non‐severe injuries and in those developing ALI 72 h after admission (Figure 3E–H). There was a similar trend for shorter fragment lengths in patients developing AKI. Some studies suggest shorter fragments may bind with higher affinity to proinflammatory nucleic acid receptors than longer fragments.^9^

**FIGURE 3:**
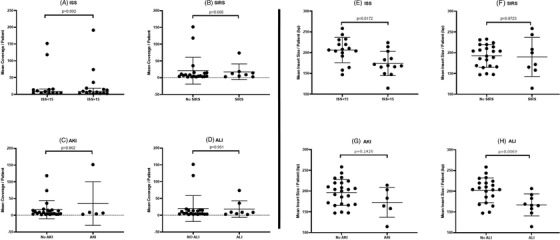
**Fragment length but not normalized coverage correlate with injury scores or severity**. (A–D) Normalized coverage determined by mean coverage/mean nuclear mitochondrial (NUMT) coverage. Systemic inflammatory response syndrome (SIRS) defined by a patient with two or more criteria for systemic inflammatory response syndrome. Acute kidney injury (AKI) was determined by the KDIGO Criterial and while acute lung injury (ALI) was assessed by a PaO_2_:FiO_2_ ratio between 100–200 in patients requiring at least 40% FiO_2_ at 72 h post‐admittance. (E–H) Insert size determined by the mean length of the mitochondrial DNA (mtDNA) fragment (bp) that was sequenced in each patient. Non‐parametric Mann‐Whitney U‐tests were performed to calculate the indicated *p*‐values for (A–H).

To determine if plasma mtDNA heteroplasmies were related to patient outcomes in this cohort of trauma patients, we next calculated odds ratios quantifying the association of all minor allele variants with the trauma‐related clinical phenotypes. A ≥25% cutoff was used to designate variants of interest to minimize the probability of including sequencing artefacts in this analysis. As depicted by Solar Manhattan plots, significant variants are associated with ISS, SIRS, AKI and ALI (Fisher's Exact test; Figure 4A–D). Most interesting was a cluster of variants associated with AKI in the D‐loop transcriptional control region, while ALI was accompanied by a significant variant in the cytochrome B region. While the number of patients analyzed is too small to draw rigorous conclusions, the results demonstrate the potential application of this protocol in a mitochondrial genome‐wide association study.

**FIGURE 4:**
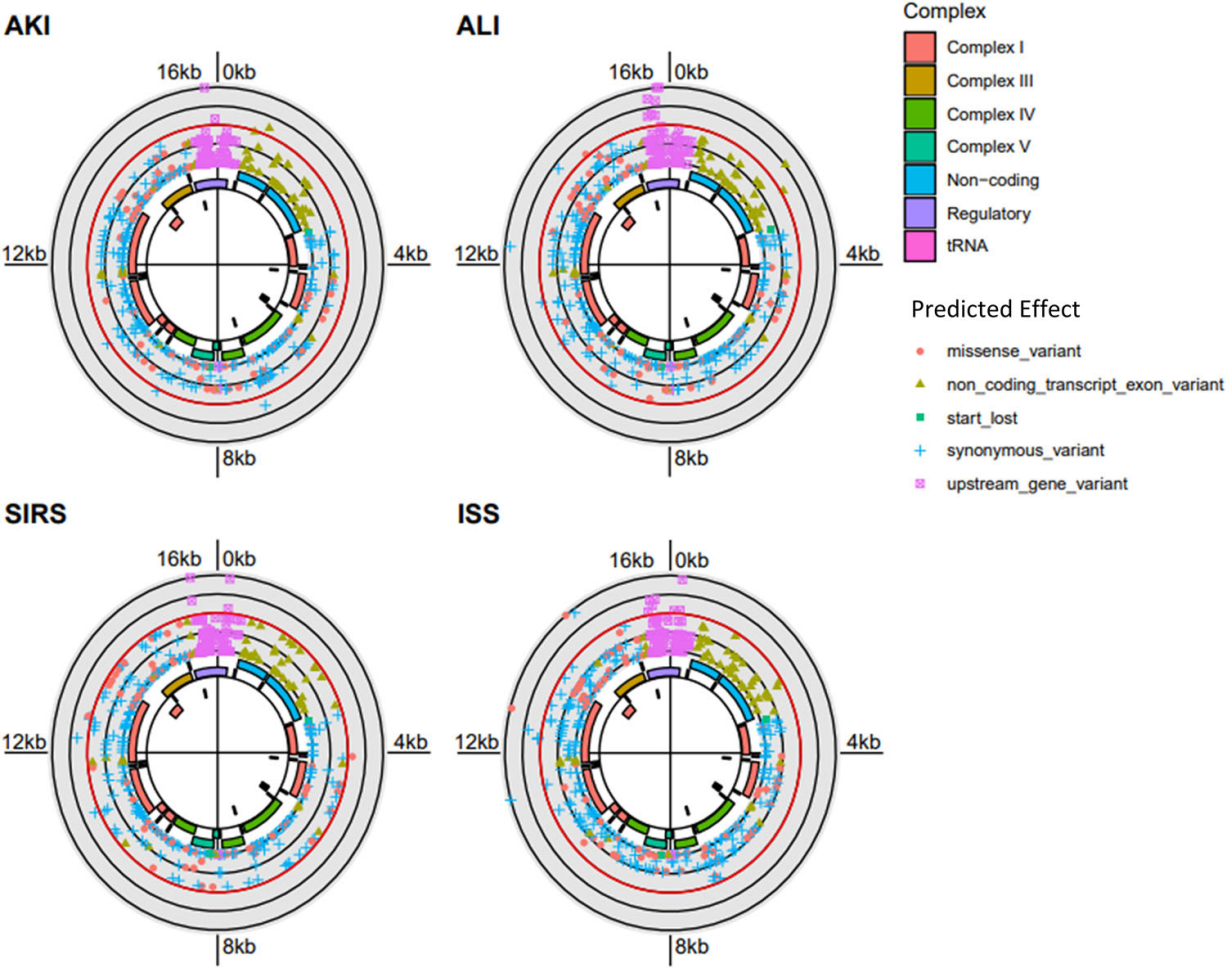
**Solar Manhattan plots depicting plasma mitochondrial DNA (mtDNA) variants associated with severe outcomes**. Variants meeting the minimum variant allele fraction density ≥25%; mtDNA region is denoted by coloured sequences. Variants significantly increased for risk of indicated complications located at or outside the red ring. Functional regions of the mitochondrial genome are displayed in the interior, and variants are coloured and shaped by *in silico* predicted effect. Acute kidney injury (AKI) was associated with multiple significant variants in the D‐loop control region while acute lung injury (ALI)‐related variants were present in D‐loop and cytochrome B regions. Odds ratios for individual variants were calculated. Associations are considered significant at *p* < .05 and are plotted as the negative log10 of the *p*‐values at each mitochondrial position (denoted in the figure by any point outside of the red circle). The figure was rendered with the ggbio R package.

In summary, here we present an experimental and analytical workflow that permits detailed evaluation of plasma cell‐free mtDNA DAMP abundance and characteristics across the entire genome with increased accuracy and sensitivity over quantitative‐PCR using commercially available reagents and open‐source analysis software. In this cohort of trauma patients, we find that shorter mtDNA fragment length may be more sensitive for predicting acute patient outcomes than mtDNA DAMP abundance. Future studies can take advantage of this strategy to explore how attributes of plasma mtDNA DAMPs predict outcomes in patients with trauma, sepsis, and multiple other diseases.

## CONFLICT OF INTEREST

The authors declare that they have no conflict of interest.

## Supporting information



Supporting Information

Tables

Figure S1

Figure S2
