# Supplementary material for: Novel attributes of cell‐free plasma mitochondrial DNA in traumatic injury
Source: Clin Transl Med. 2022 Oct 17;12(10):e1055. doi: 10.1002/ctm2.1055 (PMC9574491; doi:10.1002/ctm2.1055)
Supplement: Supplementary file 1 — Supporting Information [file CTM2-12-e1055-s002.docx]

**METHODS**

**Study subjects:** This study proceeded in two phases both approved by the University of South Alabama and University of Mississippi Medical Center IRBs, respectively. The goal of the first phase was to develop an analytical algorithm to quantify mtDNA DAMPs as well as call heteroplasmic variants. For this study, we obtained plasma from four trauma patients admitted to the Surgical/Trauma Intensive Care Unit (STICU) at the University of South Alabama Health University Hospital Trauma Center. The study was approved by the University of South Alabama Institutional Review Board. Informed written consent was obtained from the patient or legal representative prior to the samples being utilized for experimental purposes. Any samples collected which were not subsequently consented to were destroyed. All experimental protocols were approved by University Hospital, and the methods performed were in accordance with the relevant guidelines and regulations. Inclusion criteria were adults (≥18 years) that were admitted to the STICU with an Injury Severity Score (ISS) ≥15. Plasma (8 ml) was collected immediately upon admission to the STICU and 72h later. Two patients required activation of an institutional massive transfusion protocol (> 15U of blood products) while two patients with similar ISS did not require transfusion products (demographics; Supplemental Table 1). Plasma also was obtained from a human biorepository, but in this instance, mtDNA was below the limits of qPCR detection or the mtDNA present was full-length (Supplemental Fig. 2).

Enrollment of 30 patients for phase 2 of this work, whose goal was to validate the utility of the algorithm developed in phase 1, was conducted concurrently. The study was approved by the University of Mississippi Institutional Review Board. Informed written consent was obtained from the patient or legal representative prior to the samples being utilized for experimental purposes. Inclusion criteria were adults (≥18 years) meeting criteria for highest-level trauma activation based on institutional protocols. Plasma was obtained from injured patients evaluated at the University of Mississippi Medical Center Emergency Department within 6 hours of arrival. All experimental protocols and methods performed were in accordance with the relevant guidelines and regulations. Demographics are in Supplemental Table 2.

**Clinical phenotyping**: ISS was assessed based on discharge diagnoses, and the presence of SIRS, AKI and ALI assessed at 72 hours after enrollment. Patients were segregated into four clinical descriptors: 1) those with or without major trauma (ISS ≥15); 2) AKI, as determined by the KDIGO criteria, and 3) ALI, defined by a PaO2:FiO2 ratio between 100-200 in patients requiring at least 40% FiO2.

**mtDNA Library preparation from extracellular DNA in plasma and deep DNA sequencing:** Fresh plasma was centrifuged at 700g (4⁰C) for 5m. Supernatant was carefully removed by pipette to avoid aspirating the cell pellet. DNA was isolated from 200 μl of plasma using the QIAamp DSP DNA Blood Mini Kit (Qiagen, Inc, Hilden, Germany) according to manufacturer’s instructions and stored in 75μl elution buffer (EB) -80⁰C.

For DNA-seq library preparation, The KAPA Hyper Prep Kit was used following manufacturer’s instructions. Briefly, end repair and adenine tailing were combined in a single step, followed by adaptor ligation. DNA was purified 1.5X AMPure XP Beads (Beckman Coulter). Library amplification (11 cycles) was performed. DNA was purified using AMPure beads and stored 30 μl EB. To enrich mtDNA, we used MyBaits Human Global Panel mtDNA kit (Arbor Biosciences) following manufacturer’s instructions. Briefly, 7μl of library was hybridized with the RNA mtDNA baits for 40h at 55⁰C. The RNA-DNA hybridized baits were captured with Dynabeads MyOne Streptavidin C1 (Arbor Biosciences). After purification, the library was enriched with KAPA HIFI kit (KAPA Inc) for 8 cycles of PCR. Quantification was determined using the KAPA Library Quantification kit. Analysis of the libraries utilized the Bio-Rad Bioanalyzer (Experion BioRad Inc.).

Next Generation sequencing was performed for two patients by HudsonAlpha on a *HiSeq2000*. 2x50 paired reads, n=4 libraries 1.03*10^8^ ± 7.46*10^6^ reads (mean ± SD). The other two samples were sequenced at Florida State University on a *HiSeq2500* at 2x50 paired reads, n=7 libraries with 3.11*10^7^ +- 8.95*10^6 ­­^reads (mean ± SD). Four samples were run prior to enrichment to determine enrichment efficiency of the MyBaits protocol. One of the libraries failed due to low amount of isolated cfDNA; after enrichment, however, the sample contained adequate mtDNA for sequencing. For the expanded study, 30 patients were sequenced with 2x150bp paired reads, n=30 libraries 3.69*10^7^ +- 1.84*10^7^ reads (mean ± SD).

**Code Availability:** Main code for the workflow is contained at ***github.com/GrantDaly/mitochondrial-alignments***. Code for variant calling is contained at ***github.com/GrantDaly/bcftools***. The workflow runner employed was the *Broad Institute’s* *Terra* platform (https://app.terra.bio/), and *Dockstore* was used to manage the WDL workflows employed^1,2^.

**Alignments:** Primary processing was performed with a *Docker* image “gdaly9000/mitochondrial”, available from *Dockerhub*. The image contained samtools 1.14-3-gd85a610 with htslib 1.14-1-g7060387, *Bcftools* 1.12-32-g5f124d6, and *Cutadapt* v3.4^3-5^. Adapter trimming was performed with *Cutadapt*. Paired-end reads were aligned to the GRCh38 reference assembly with *BWA-MEM (MD5sum 21e2e08d7fc1bf7b88f67cb369039642)^6,7^*. Samtools “fixmate” was used to ensure reads had correct mate pair information and *Samtools* “sort” was used to sort reads by position.

**Nuclear Mitochondrial Insertions:** The previously annotated reference NUMT set available had originally been generated with the outdated *hg18* assembly, so we elected to generate a NUMT call-set for the current GRCh38 assembly^8^. *NCBI Nucleotide Blast 2.6.0* was run on the *Alabama Supercomputer* with the query being the human mitochondrial sequence, database GRCh38, word size 9, reward 1, penalty -1, gapopen 2, and gapextend 2^9^. This resulted in 1521 NUMT covering 1.03*10^5^bp. This BLAST-derived set will be referred to as “BLAST NUMT.”

Polymorphic NUMTs are more recent insertions not found in the reference assembly, but are instead structural population variants. A “vcf” file retrieved from Dayama et al.^10^ provided coordinates for the 95% CI of the insertion sites, and *Bedtools v2.26.0* “slop” added 200bp upstream and downstream of the insertion sites^11^. GRCh37 coordinates were then converted to GRCh38 coordinates with the UCSC *Liftover* utility, with 138/141 sites successfully lifting over^12^.

**Validation of Alignment Pipeline by Simulation:** To evaluate the effect of NUMTs on the accuracy of our alignment pipeline, a read simulation method was employed. First, *Bedtools v2.26.0* “slop” added 100 bp flanking regions upstream and downstream to the previously described non-polymorphic NUMTs. Then *Bedtools* “merge” merged any of these intervals within 100 bp, which resulted in 699 remaining intervals. Using fragment size parameters derived from one of our samples, we used *Art Illumina Q Version 2.5.8* to simulate reads with the error model of a *HiSeq 2500* instrument, read length 50bp and 150bp, insert size 100bp and 150bp ± 25 (mean ± StDev). Reads were simulated for the mitochondrial sequence (with the “N” spacer character removed from position 3107), the merged NUMT sequences, and the known polymorphic NUMT sequences^10,13^. Coverage was parameterized as 1,000 for the mitochondrial and NUMT reads. Simulated reads were processed through our standard alignment and coverage pipeline.

**Empirical NUMT as Proxy for Nuclear DNA Quantity:** To use NUMT as a stand-in for nuclear DNA copy number, we identified regions which not only shared homology with the mitochondrial genome, but could be enriched by the bait capture kit and aligned to the human genome using our alignment workflow. The *Deeptools* v3.5.1 bamcompare utility was used to call coverage for simulated mitochondrial data for 50bp reads and 100 bp inserts, and *iSeg v0.2* (program available upon request) was used to call peaks^14,15^. These simulated peaks were combined with the *ENCODE* blacklist v2 (github.com/Boyle-Lab) to form a blacklist of unreliable regions^16^. *Deeptools* bamCompare was used to calculate enrichment between the WGS replicate and bait-capture replicate for the three samples, using a minimum mapping quality 20. *iSeg* was used to call peaks, and *Bedtools* *v2.26.0* intersect was used to select peak regions shared by all three replicates. This set was labeled “Empirical NUMT”.

**Mitochondrial Coverage and Insert:** The in-house tool inserts-and-cov (github.com/GrantDaly/mitochondrial-alignments) was used to calculate coverage over the mitochondria and NUMTs. The program identifies fragments which map to the forward or reverse strand and records the total number of bases of the reference genome covered by the fragment. The program requires read mapping quality of at least 20. Forward and reverse genome coverage are recorded, and total coverage is the sum of these numbers at any given locus. The tool also calculates insert size statistics, selecting read ones with mapping quality ≥20, and which are between one and 1000 bp in length. Mitochondrial coverage is then normalized to nuclear coverage by dividing mitochondrial coverage by the mean NUMT coverage of all NUMT (Mitochondrial Coverage / NUMT).

**Variant Calling:** A Bcftools plugin “heteroplasmy” was created in a forked GitHub repository (github.com/GrantDaly/bcftools). To call a homoplasmic variant, an allele needed to have 10 bases on the forward and reverse strand and have a variant allele fraction (VAF) at least 0.99. Heteroplasmic variants were called when an allele had at least 10 bases in the forward and reverse strand, but additionally required at least 500 base coverage and an minor allele fraction of ≥ 0.01. Bedtools “subtract” was used to remove variants in regions marked by “blacklist_sites.hg38.chrM.bed” retrieved from the Broad Institute’s public *Terra* workflow “Mitochondria-SNPs-Indels-hg38”. Bcftools “norm” was then used to convert the vcf format file into a tsv format file for downstream analysis. For downstream analysis only heteroplasmies with VAF ≥0.05 were considered to further rule out the possibility of sequencing or alignment artifacts, as well as to only examine heteroplasmies likely to be physiologically relevant. Finally, ENSEMBL variant effect prediction and variation APIs were called to add annotations to the variants^17^.

**Raw Data Files:** We calculated mean coverage for total mtDNA, NUMTs, and normalized coverage (Supplemental Table S3, S4). Normalized mtDNA coverage was additionally called over each base of the mitochondrial genome (Supplemental Table S7). Coverage statistics for reference NUMTs are included in Supplementary Table S8. We also calculated mean coverage at 138 previously identified polymorphic NUMT insertions to determine patient-specific associations (Supplementary Table S9). Insert size statistics are included in Supplemental Table S5. Heteroplasmic variants as determined by the variant calling method described above are included in Supplemental Table S10.

**Statistics:** Most figures were made using *Graphpad Prism* 7.0 (GraphPad Software, San Diego, CA, USA). Of particular importance were Python v3.7.9 (<http://www.python.org/>) executed in a Jupyter Notebook, Pandas and Numpy data analysis libraries, and Matplotlib and Seaborn plotting libraries to plot the fragment size distributions^18-24^ *Inkscape 1.0.2* was used for one diagram^25^.

**Solar Manhattan Plots**: Variants with VAF ≥ 25% were selected and the *Python* “Scipy” library’s Fisher’s exact test with alternative “greater” was tested the association between specific variants and ISS, SIRS, AKI, and ALI, and p-values and odds ratios of significant hits were recorded in Supplemental Table S6^26^. The *ggbio R* package was used to plot the -log10(p-value) for these variants^27^.

**DATA AVAILABILITY**

Raw sequencing data for phase 1 data are available from the Sequence Read Archive accession PRJNA727684. Data for phase 2 are available upon request.

**REFERENCES**

1. O'Connor BD, Yuen D, Chung V, et al. The Dockstore: enabling modular, community-focused sharing of Docker-based genomics tools and workflows. F1000Res 2017;6:52. DOI: 10.12688/f1000research.10137.1.

2. Voss K, Auwera G, Gentry J. Full-stack genomics pipelining with GATK4 + WDL + Cromwell. 2017. DOI: 10.7490/F1000RESEARCH.1114631.1.

3. Danecek P, Bonfield JK, Liddle J, et al. Twelve years of SAMtools and BCFtools. Gigascience 2021;10(2). DOI: 10.1093/gigascience/giab008.

4. Martin M. Cutadapt removes adapter sequences from high-throughput sequencing reads. EMBnetjournal 2011;17(1):3.

5. Merkel D. Docker: lightweight linux containers for consistent development and deployment. Linux journal 2014;2014(239):2.

6. Schneider VA, Graves-Lindsay T, Howe K, et al. Evaluation of GRCh38 and de novo haploid genome assemblies demonstrates the enduring quality of the reference assembly. Genome Res 2017;27(5):849-864. DOI: 10.1101/gr.213611.116.

7. Li H. Aligning sequence reads, clone sequences and assembly contigs with BWA-MEM. arXiv; 2013.

8. Simone D, Calabrese FM, Lang M, Gasparre G, Attimonelli M. The reference human nuclear mitochondrial sequences compilation validated and implemented on the UCSC genome browser. BMC Genomics 2011;12:517. DOI: 10.1186/1471-2164-12-517.

9. Camacho C, Coulouris G, Avagyan V, et al. BLAST+: architecture and applications. BMC Bioinformatics 2009;10:421. DOI: 10.1186/1471-2105-10-421.

10. Dayama G, Emery SB, Kidd JM, Mills RE. The genomic landscape of polymorphic human nuclear mitochondrial insertions. Nucleic Acids Res 2014;42(20):12640-9. DOI: 10.1093/nar/gku1038.

11. Quinlan AR. BEDTools: The Swiss-Army Tool for Genome Feature Analysis. Curr Protoc Bioinformatics 2014;47:11 12 1-34. DOI: 10.1002/0471250953.bi1112s47.

12. Kent WJ, Sugnet CW, Furey TS, et al. The Human Genome Browser at UCSC. Genome Research 2002;12(6):996-1006. (In en). DOI: 10.1101/gr.229102.

13. Huang W, Li L, Myers JR, Marth GT. ART: a next-generation sequencing read simulator. Bioinformatics 2012;28(4):593-4. DOI: 10.1093/bioinformatics/btr708.

14. Ramírez F, Ryan DP, Grüning B, et al. deepTools2: a next generation web server for deep-sequencing data analysis. Nucleic Acids Research 2016;44(W1):W160-W165. (In en). DOI: 10.1093/nar/gkw257.

15. Girimurugan SB, Liu Y, Lung PY, et al. iSeg: an efficient algorithm for segmentation of genomic and epigenomic data. BMC Bioinformatics 2018;19(1):131. DOI: 10.1186/s12859-018-2140-3.

16. Amemiya HM, Kundaje A, Boyle AP. The ENCODE Blacklist: Identification of Problematic Regions of the Genome. Sci Rep 2019;9(1):9354. DOI: 10.1038/s41598-019-45839-z.

17. McLaren W, Gil L, Hunt SE, et al. The Ensembl Variant Effect Predictor. Genome Biology 2016;17(1):122. DOI: 10.1186/s13059-016-0974-4.

18. Kluyver T, Ragan-Kelley B, Pérez F, et al. Jupyter Notebooks – a publishing format for reproducible computational workflows. In: Loizides F, Schmidt B, eds.: IOS Press; 2016:87 - 90.

19. McKinney W. Data Structures for Statistical Computing in Python. Python in Science Conference2010:56-61.

20. Reback J, McKinney W, jbrockmendel, et al. pandas-dev/pandas: Pandas 1.1.3. v1.1.3 ed: Zenodo; 2020.

21. Harris CR, Millman KJ, Walt SJvd, et al. Array programming with NumPy. Nature 2020;585(7825):357–362. DOI: 10.1038/s41586-020-2649-2.

22. Hunter JD. Matplotlib: A 2D graphics environment. Computing in Science & Engineering 2007;9(3):90–95. DOI: 10.1109/MCSE.2007.55.

23. Waskom ML. seaborn: statistical data visualization. Journal of Open Source Software 2021;6(60):3021. DOI: 10.21105/joss.03021.

24. Van Rossum G, Drake FL. Python 3 Reference Manual. Scotts Valley, CA: CreateSpace, 2009.

25. Project I. Inkscape. 0.92.5 ed2020.

26. Virtanen P, Gommers R, Oliphant TE, et al. SciPy 1.0: Fundamental Algorithms for Scientific Computing in Python. Nature Methods 2020;17:261–272. DOI: 10.1038/s41592-019-0686-2.

27. Yin T, Cook D, Lawrence M. ggbio: an R package for extending the grammar of graphics for genomic data. Genome Biol 2012;13(8):R77. DOI: 10.1186/gb-2012-13-8-r77.
