# Supplementary figures and images for: Novel attributes of cell‐free plasma mitochondrial DNA in traumatic injury
Source: Clin Transl Med. 2022 Oct 17;12(10):e1055. doi: 10.1002/ctm2.1055 (PMC9574491; doi:10.1002/ctm2.1055)

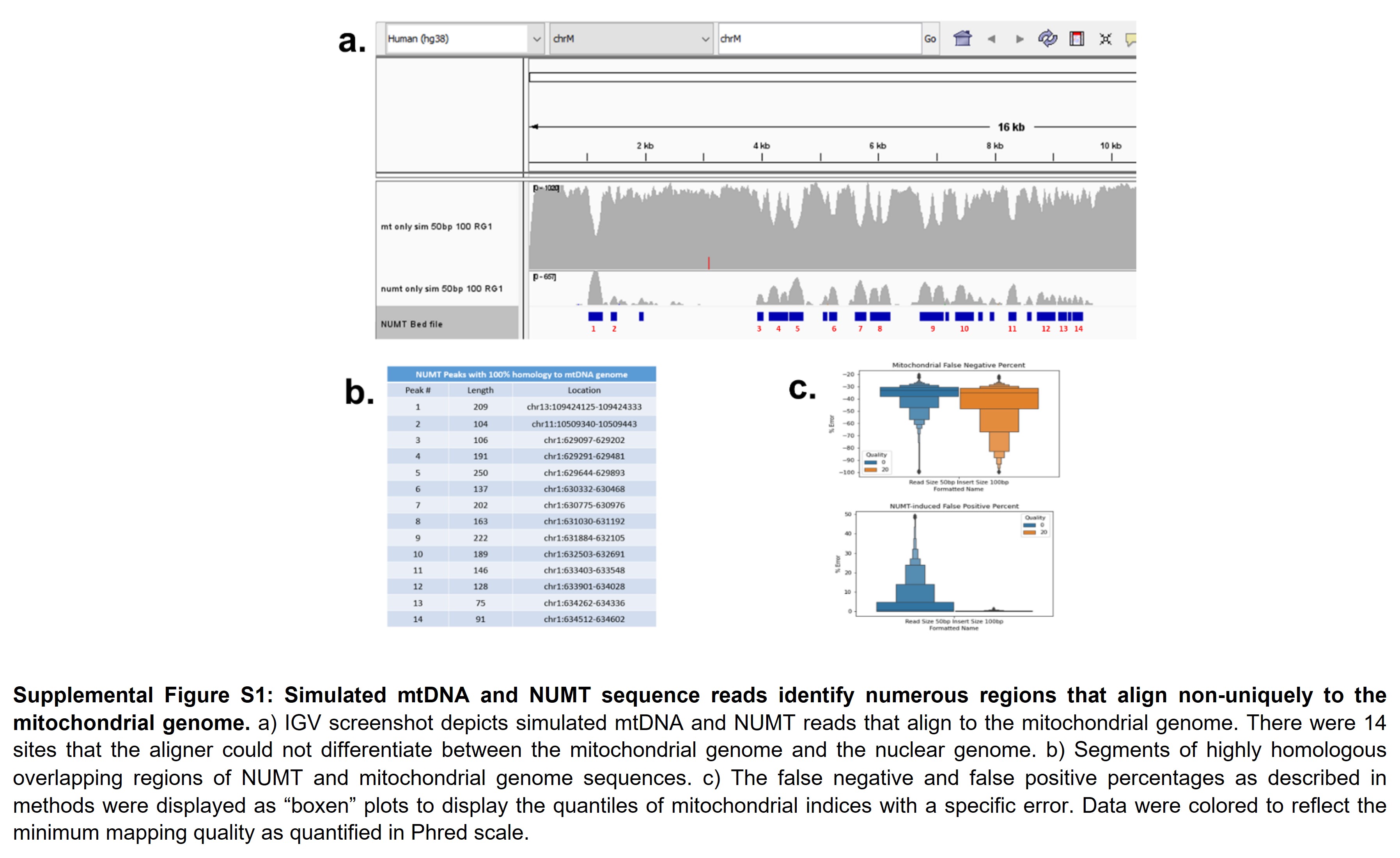

Supplement: Supplementary file 3 — Figure S1 [file CTM2-12-e1055-s003.jpg]

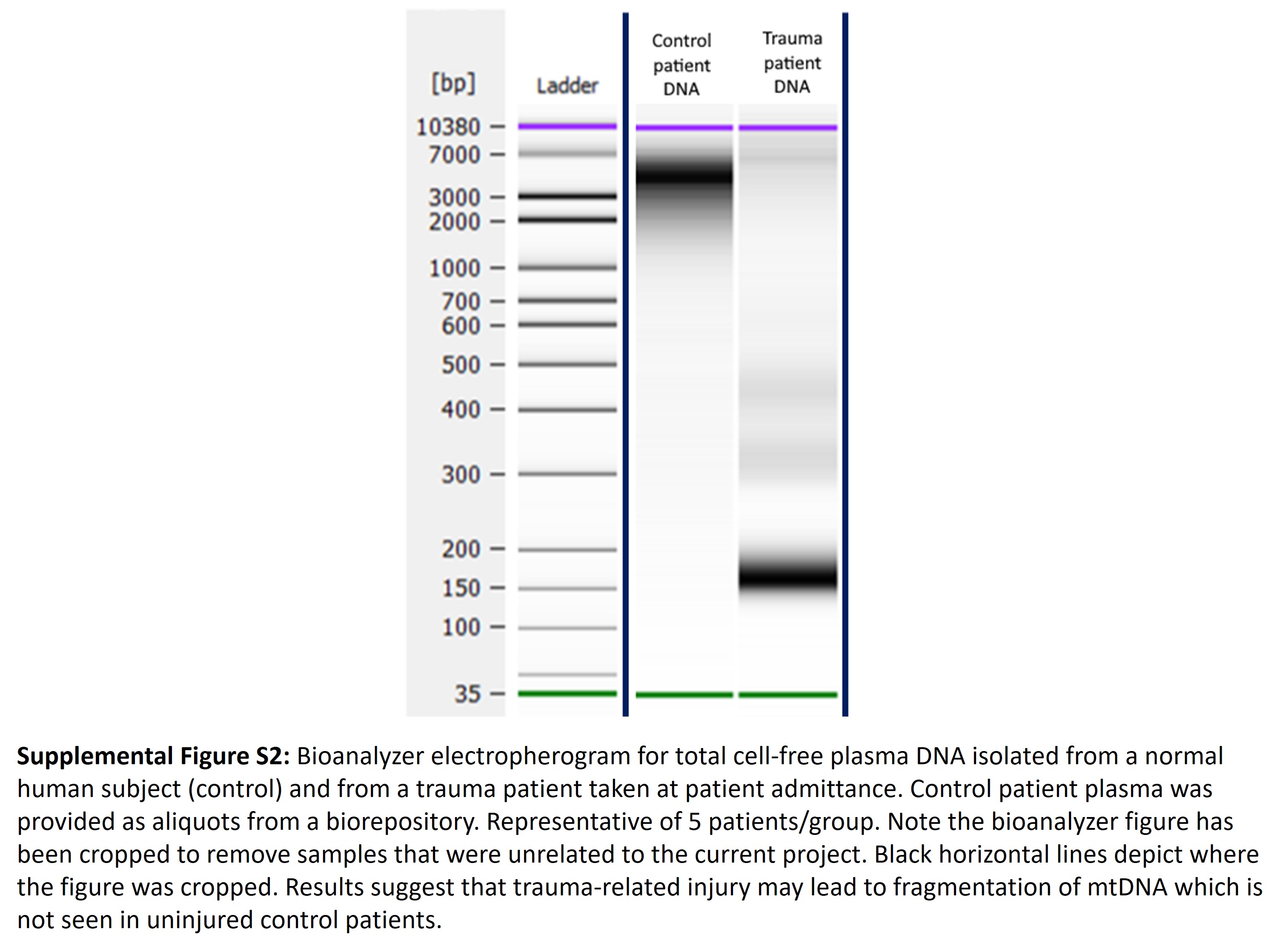

Supplement: Supplementary file 4 — Figure S2 [file CTM2-12-e1055-s001.jpg]
